# Une tumeur neuroectodermique primitive périphérique à localisation gastrique primaire: à propos d’un nouveau cas

**Published:** 2010-08-20

**Authors:** Ankouz Amal, Elbouhadouti Hicham, Lamrani Jihane, Bouassria Abdesslam, Louchi Abdelatif, taleb khalid Ait

**Affiliations:** 1 Service de chirurgie viscérale A, CHU Hassan II de Fès, Maroc

**Keywords:** Tumeur neuroectodermique primitive périphérique, pPNET, Gastrique

## Abstract

**Abstract:**

Les tumeurs neuro-ectodermiques primitives ou sarcome d’Ewing sont classiquement des néoplasmes se développant aux dépends des tissus mous et des os. Les tumeurs neuro-ectodermiques primitives gastriques (pPNETs) sont extrêmement rares. Nous nous proposons, à travers le cas d’un patient, opéré pour une tumeur gastrique, d’étudier les aspects cliniques, radiologiques, anatomopathologiques et thérapeutiques des tumeurs neuro-ectodermiques primitives périphériques. A notre connaissance ce patient illustre le troisième cas de tumeur gastrique d’origine neuroectodermique décrite chez l’adulte.

## Introduction

Les tumeurs neuro-ectodermiques primitives appartiennent à la famille des tumeurs sarcomateuses d’Ewing qui dérivent toutes de la même cellule souches [1]. Les cellules de la crête neurale sont soupconnées d’être à l’origine de ces tumeurs. Les sites de prédilections de ces sarcomes sont: la région paravertébrale, la paroi thoracique et les extrémités distales. Plus rarement, ils se développent aux dépens du poumon, de l’utérus, des ovaires, des voies urinaires, du myocarde, de la glande parotide et du rein [1,2]. Le diagnostic positif des tumeurs neuro-ectodermiques primitives nécessite la contribution de l’histopathologie, de l’immunohistochimie et de la cytogénétique.

## Patient et observation

**Figure 1:**
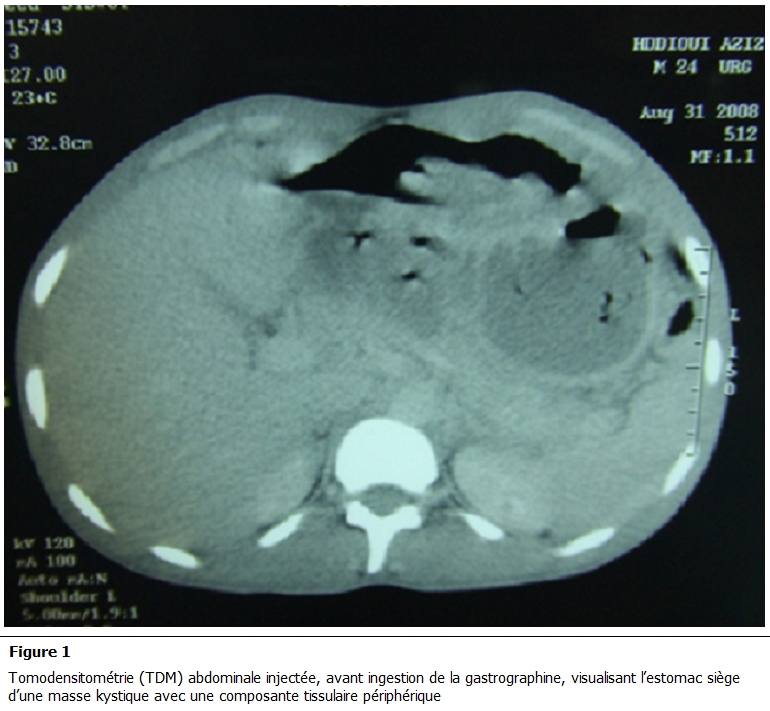
Tomodensitométrie (TDM) abdominale injectée, avant ingestion de la gastrographine, visualisant l’estomac siège d’une masse kystique avec une composante tissulaire périphérique.

Il s’agit d’un homme de 30 ans, sans antécédents particuliers, se plaignant de douleurs épigastriques évoluant depuis un mois dans un contexte d’apyrexie et de conservation de l’état général, sans vomissement ni hémorragie digestive extériorisée. L’examen clinique a objectivé une masse épigastrique de 9 cm de grand axe fixée au plan profond mobile par rapport au plan superficiel et indolore. La fibroscopie oesogastroduodénale a révélé la présence d’une ulcération antropylorique de la face antérieure de 2 cm de grand axe. L’échographie abdominale a montré l’existence d’une masse tissulaire de 10 cm de grand axe, présentant des végétations endokystiques et se développant au dépend de l’estomac sans ascite et sans image de métastase hépatique. La Tomodensitométrie (TDM) abdominale a confirmé ces données, et a visualisé la tumeur kystique avec sa composante tissulaire périphérique rehaussée par le produit de contraste (Figure 1 et 2).

**Figure 3:**
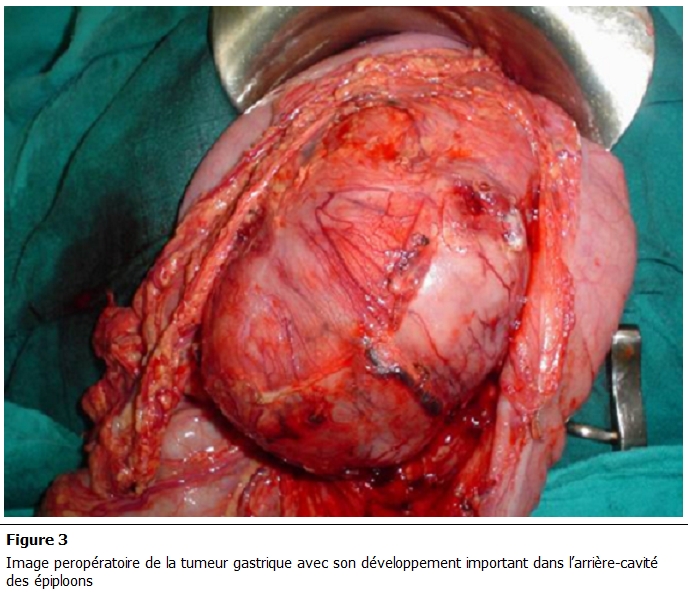
Image préopératoire de la tumeur gastrique avec son développement important dans l’arrière-cavité des épiploons.

Biologiquement, le taux de l’antigène carcino-embryonnaire était élevé à 9 Lg/l. Le bilan d’extension réalisé ainsi que le bilan préopératoire étaient sans particularités. Une gastrectomie totale, emportant la totalité de la tumeur, avec curage ganglionnaire ont été réalisés (Figure 3).

Le rétablissement de la continuité a été réalisé au moyen d’une anastomose oeso-jéjunale sur anse en Y. Les suites opératoires ont été simples avec reprise de l’alimentation au 5^ème^ jour. L’examen anatomopathologique de la tumeur trouvait des massifs de petites cellules contenant des tâches noirâtres, des nucléoles arrondis et ovales, un cytoplasme éosinophile et des mitoses fréquentes. Ces cellules présentaient une discrète différentiation en rosette et un stroma de nature desmoplastique. Les ganglions n’étaient pas envahis. L’immunomarquage par les anticorps anticytokératine, antichromagranine, antidésmine et antisynaptophysine était négatif. En revanche, celui par l’anticorps anti-CD99 (Mic2) était fortement positif. Il s’agissait donc, d’une localisation gastrique d’une tumeur neuroectodérmique primitive périphérique. Le geste chirurgical étant jugé curatif, le complément chimiothérapique n’a pas été indiqué. Au recul de six mois, le contrôbiologique et scanographique n’a pas objectivé de récidive.

## Discussion

En janvier 2000, l’OMS a classé Les pPNET comme appartenant au groupe des tumeurs neuroépithéliales embryonnaires c’est-à-dire développées à partir de cellules germinales du tube neural [3-5]. Ces tumeurs représentent moins de 1 % de tous les sarcomes. Elles prédominent avant l’âge de 35 ans (75 % des cas) avec un pic de fréquence entre 15 et 20 ans. Neuf cas sur dix sont de race blanche avec une légère prédominance masculine, mais aucun facteur favorisant n’est retrouvé [6]. La localisation thoracique est la plus fréquente (44%) suivie des pPNET abdominopelviens (26%) puis des extrémités (20 %) et enfin de la tête (6 %) [1].

A notre connaissance, notre cas serait le 3ème cas de pPNET gastrique décrit chez l’adulte [7,8]. Les signes cliniques manquent de spécificité. La douleur abdominale ainsi que la présence d’une masse abdominale et d’une fièvre sont les symptô les plus fréquents. La biologie est également non spécifique. Les examens radiologiques explorent la tumeur et recherchent les métastases, localisées par ordre de fréquence aux poumons (50 %), os (25 %), moelle (20 %), foie et cerveau [2], jugeant ainsi de la résécabilité de la tumeur primitive. D’un point de vue anatomopathologie, la tumeur est multilobulaire, molle, friable. Elle est en général blanchâtre avec de larges plages de nécroses, des formations kystiques et des hémorragies intratumorales [1]. D’un point de vue microscopique, les cellules tumorales sont rondes, avec un noyau arrondi. La membrane nucléaire est bien distincte, avec une chromatine fine et parfois un ou deux petits nucléoles. Les mitoses sont plus ou moins fréquentes. En général on note la présence de grains de glycogène intracytoplasmiques. La présence de rosettes ou pseudo-rosettes caractérise plus précisément les PNET. La plupart du temps, ces tumeurs surexpriment un antigène déterminé par le gène MIC2 désigné sous le nom de CD99. Elles surexpriment d’autre part une autre protéine appelée FLI1 issue de la translocation entre le chromosome 22 et le chromosome 11.

Le diagnostic de pPNET repose sur la mise en évidence d’un transcrit par technique de génétique moléculaire à savoir une RT-PCR (rétrotranscription- polymerase chain reaction) [2,9-11]. Le traitement des PPNET est lourd et dépend de la localisation, du grade histologique, et de la présence ou non de métastases. Il inclut dans la majorité des cas une chirurgie radicale, une chimiothérapie néo-adjuvante et/ou adjuvante et une radiothérapie.

Concernant notre cas, le traitement était uniquement chirurgical. La chirurgie réglée garde une place prépondérante, surtout dans les formes localisées. Les pPNET sont connues d’être chimio-sensibles surtout celles résultant du transcript EWS/FLI1 et présentant une prolifération tumorale plus lente. Cette chimiosensibilité est prouvée suite à la réponse presque complète de 94% de patients traités par vincristine, adriamycine et cyclophosphamide [12]. La chimiothérapie néoadjuvante est préférable à l’adjuvante car elle permet d’évaluer la réponse tumorale au temps chirurgical et de décider de l’intensification éventuelle ou d’un complément radiothérapeutique.

La surveillance postopératoire est à la fois clinique et radiologique et se fait tous les trois mois sur trois ans puis tous les six mois sur deux ans et enfin tous les ans. La récidive tumorale est plus fréquente durant les dix premières années. En cas de marge de résection insuffisante ou envahie, ou de forme métastasée, la radiothérapie devient nécessaire. Cette dernière est délivrée à une dose variant de 45 à 56 Grays et son fractionnement est site dépendant [6,13].

## Conclusion

L’arsenal incluant une chirurgie de résection, une chimiothérapie néoadjuvante et adjuvante et une radiothérapie première, constitue actuellement le traitement de choix des pPNET. Cependant le diagnostic préopératoire est souvent difficile. La survie reste médiocre ; elle varie entre 28-38% à 2 ans et chute à 14-17% à 6 ans [14].

## Conflits d’intérêt

Les auteurs déclarent n’avoir aucuns conflits d’intérêts.

## Contribution des auteurs

AA : a rédigé l’article et à contribuer à la prise des photos. HE, JL et AB ont contribué à la recherche bibliographique. AL et KA ont assuré la prise en charge thérapeutique du malade et ont contribué à la rédaction de ce document.

## Consentement

Les auteurs déclarent avoir recu le consentement écrit du patient pour reporter ce cas.

## Figures and Tables

**Figure 2:**
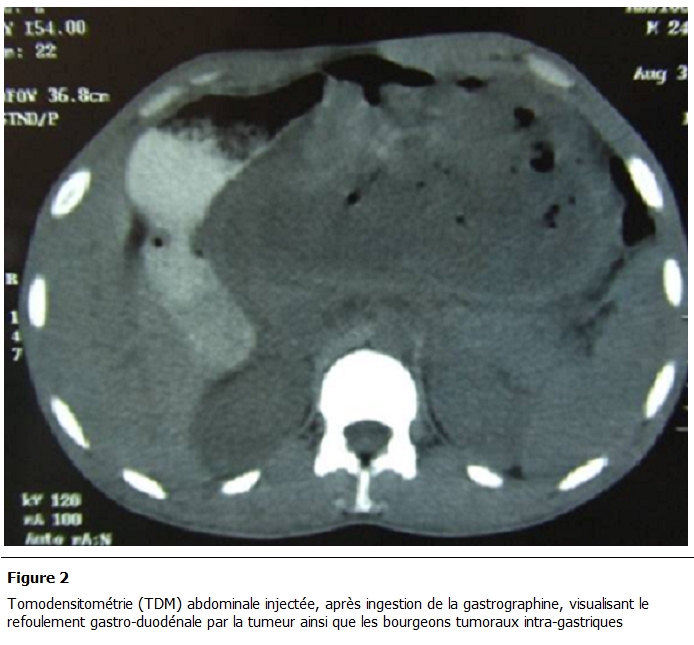
Tomodensitométrie (TDM) abdominale injectée, après ingestion de la gastrographine, visualisant le refoulement gastro-duodénale par la tumeur ainsi que les bourgeons tumoraux intra-gastriques.
